# Dynamics of Dual Scale-Free Polymer Networks

**DOI:** 10.3390/polym9110577

**Published:** 2017-11-04

**Authors:** Mircea Galiceanu, Luan Tota de Carvalho, Oliver Mülken, Maxim Dolgushev

**Affiliations:** 1 Departamento de Fisica, Universidade Federal do Amazonas, Manaus 69077-000, Brazil; mircea@ufam.edu.br (M.G.); luantota74@gmail.com (L.T.d.C.); 2 Institute of Physics, University of Freiburg, Hermann-Herder-Str. 3, 79104 Freiburg, Germany; muelken@physik.uni-freiburg.de

**Keywords:** polymer networks, scale-free networks, mechanical relaxation, eigenvalue problem

## Abstract

We focus on macromolecules which are modeled as sequentially growing dual scale-free networks. The dual networks are built by replacing star-like units of the primal treelike scale-free networks through rings, which are then transformed in a small-world manner up to the complete graphs. In this respect, the parameter γ describing the degree distribution in the primal treelike scale-free networks regulates the size of the dual units. The transition towards the networks of complete graphs is controlled by the probability *p* of adding a link between non-neighboring nodes of the same initial ring. The relaxation dynamics of the polymer networks is studied in the framework of generalized Gaussian structures by using the full eigenvalue spectrum of the Laplacian matrix. The dynamical quantities on which we focus here are the averaged monomer displacement and the mechanical relaxation moduli. For several intermediate values of the parameters’ set (γ,p), we encounter for these dynamical properties regions of constant in-between slope.

## 1. Introduction

Nowadays, in different areas of science, such as physics, chemistry, biology, economics, the study of complex networks becomes of huge significance. In particular, the concept of scale-free networks was applied with great success to the World Wide Web [1,2], metabolic networks in biological organisms [3], reaction–diffusion processes [4], financial networks [5], and transport networks [6,7], to name only a few, but also to model hyperbranched polymers [8,9]. Inspired by recent experimental techniques allowing chemical transformations to be made from hyperbranched polymers to functional core–shell nanogel systems [10], and due to our interest in the fundamental role of the presence of loops in polymer networks (e.g., in crosslinked systems [11], elastomers [12], and porous materials [13,14]), we study in this article a new kind of polymer network—the dual scale-free networks.

Here we construct dual scale-free polymers by using the procedure implemented in reference [15]. Being the dual structures [16] of treelike scale-free networks, which have a power-law distribution for their degrees [4,9,17,18], our networks contain dual units with their sizes following the same power-law decay. The limiting topologies that one can get as a function of this power-law exponent, γ, are networks made of huge dual units for very low values of γ and linear chains for very high values of γ. For intermediate values, we obtain networks composed of dual units of diverse sizes, coupled one to another. In our model, the minimum allowed size corresponds to a line (or two connected nodes), which guarantees that the construction procedure never stops by itself, but only when the desired network’s size is reached. The dual units considered in this article range from rings to complete graphs. The transition between these units is implemented by adding, with probability *p*, links between nodes from the same ring. In this way, we get the ring limit for p=0 and for p=1 we obtain complete graphs. The relaxation dynamics of these networks is studied in the framework of generalized Gaussian structures (GGSs) [9,19,20,21,22,23,24,25,26,27], which concentrates on the role of structure connectivity. In the model, the monomers are visualized as beads experiencing viscous friction connected only to their nearest neighbours by means of elastic springs. The relaxation dynamics of polymers is completely determined by knowing all eigenvalues and eigenvectors of the connectivity (Laplacian) matrix, which allows one to study very large systems.

The paper is structured as follows: In Section 2 we briefly describe the algorithm used to construct the dual scale-free networks. In Section 3 we recall the general formalism of GGS and we review the basic equations which govern monomer displacement under a constant force and the mechanical relaxation of polymers. In Section 4 we study the relaxation dynamics of polymers modeled in Section 2. Here, we study the eigenvalues spectrum of our networks and then we focus on their dynamics by exploring the parameter set (γ,p). Section 5 concludes this paper.

## 2. Sequentially Growing Dual Scale-Free Networks

The original model of scale-free networks proposed by Barabási and Albert [17] attracted a lot of interest from the scientific community, also proved by the continuously increasing number of scale-free models (e.g., [4,8,9,18,28], to cite only a few). In this article we extend these works by studying the dual structures of treelike scale-free networks, making use of the model developed in reference [15] for the study of quantum transport.

The scale-free network models consider a power-law for the distribution of functionalities (or degrees):

(1) $${\bar{p}}_{k}\propto k^{-\gamma },$$

where ${\bar{p}}_{k}$ is the probability that the functionality of a node is *k* and γ is the parameter which controls how densely a network is connected. In this article, we construct scale-free networks by following the algorithm developed elsewhere [9], but differently from this reference here we consider their dual structures. This means that instead of adding a node with functionality *k* we construct a ring or a ring with additional bonds (à la small-world network [23,29,30]) consisting of *k* nodes. Equation (1) holds starting only from k=2, assuming that $p_{1}=0$. Thus, the probability of having an object with *k* nodes (for k=2 it is a bond connecting two beads, for k=3 it is a ring of three bonds) is given by

(2) $$p_{k}=\frac{k^{-\gamma }}{\sum_{j=2}^{N-1}j^{-\gamma }},$$

where *N* is the total number of nodes and the sum on the denominator is used to keep the total probability equal to 1.

**Figure 1:**
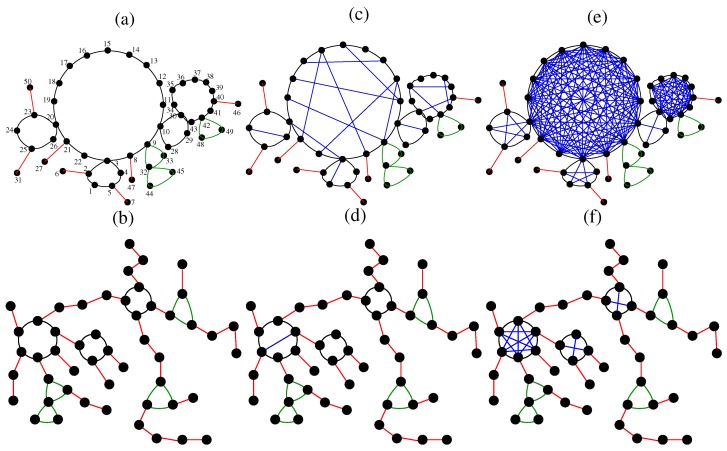
Some realizations of sequentially growing dual scale-free networks with N=50 nodes and (**a**,**b**) p=0 (ring-based dual scale-free network, rDSFN), (**c**,**d**) p=0.1 (partially dual scale-free network, pDSFN), and (**e**,**f**) p=1 (complete-graph based dual scale-free network, cDSFN). The upper row (**a**,**c**,**e**) corresponds to networks with γ=2.5, and the lower row (**b**,**d**,**f**) is for γ=4.0.

In the left column of Figure 1 we display two particular realizations of the algorithm for creating ring-based dual scale-free networks (rDSFNs) containing N=50 nodes and γ=2.5 and 4.0, from top to bottom. In order to help the reader to distinguish how the parameters’ set (γ,p) influences the topology of the network, we display by red colour the bonds (links) appearing with probability $p_{2}$ and by green colour the bonds that compose a ring of length 3. In the following, we describe the construction algorithm using Figure 1a, γ=2.5, as toy-model. In this subfigure, the numbering is according to the chronological order in which the nodes were created. The algorithm starts by randomly choosing the size of the ring from the degree-distribution (2). In this case, the first chosen size was k=5; thus, we create five nodes, labeled 1, 2, 3, 4, 5. Then, we pick at random one of the open vertices; in this case, all five nodes are still available. It turned out to be node 2, and its size was again obtained from the degree-distribution $p_{k}$. For this particular realization, the size was chosen to be k=2; thus, we have to add a line connecting node 2 with a new node, labeled by 6. This procedure is iterated until the desired network’s size is reached (N=50 for this example). The minimum allowed size is two; thus, the construction will never stop by itself since we will always have at least one open node. Additionally, by comparing Figure 1a,b one can clearly notice a transition from networks with few but large rings (low γs), to networks with many linear spacers and small rings (high γs).

In this article we focus particularly on a transition from rDSFNs to complete-graph based dual scale-free networks (cDSFNs) by performing bond percolation between non-neighboring beads of each ring inside an rDSFN. For this we introduce a new parameter, *p*, which is the probability of adding an internal bond between two non-neighboring nodes from a ring. Being a probability, the parameter *p* takes values from 0 to 1. In Figure 1c,d we show realizations of sequentially growing partially dual scale-free networks (pDSFNs) with p=0.1. The construction of these networks starts from an rDSFN with a given γ and where we add links between non-neighboring nodes of the same ring with probability p=0.1. These additional internal links are displayed by blue colour in Figure 1. In Figure 1e,f we show the complete-graph-based dual scale-free networks (cDSFNs), for which all possible internal links were added, p=1.0. We observe that by increasing γ the number of possible additional links diminishes, due to a higher amount of rings with sizes smaller than 4. This fact has a tremendous influence on the results, as will be shown in Section 4.

## 3. Theoretical Model

In this paper we study the relaxation dynamics of polymers constructed by implementing an algorithm described in the previous section. The dynamics is solved using the concept of generalized Gaussian structures (GGSs) [19,20,27,31,32], which are extensions to complex topologies of the Rouse model initially developed for linear polymer chains [33]. This model allows many features related to polymer dynamics to be studied with a very good performance, although it neglects important interactions (e.g., the hydrodynamic interactions) or sometimes essential effects (e.g., the excluded volume, the entanglements, or the stiffness). The GGS consists of *N* beads, attached to each other by Gaussian elastic springs (i.e., obeying a Gaussian statistics) with elasticity constant *K*. Here we consider the simplest case; namely, a homogeneous situation in which all the beads experience the same friction constant ζ with respect to the surrounding medium. The configuration of the GGS is given by a set of position vectors $\left\{R_{n}\right\}$, where $R_{n}\left(t\right)=\left(X_{n}\left(t\right),Y_{n}\left(t\right),Z_{n}\left(t\right)\right)$ is the position vector of the *n*th bead at time *t*. The linear Langevin equation for the dynamics of bead *i* written only for one component reads [27,31]:

(3) $$\zeta \frac{\partial Y_{i}\left(t\right)}{\partial t}+K\sum_{j=1}^{N}A_{ij}Y_{j}\left(t\right)=f_{yi}\left(t\right)+F_{yi}\left(t\right).$$

In the last equation, the friction constant can be written as ζ=6πρa, where *a* is the effective radius and ρ is the viscosity of the solvent, and the elasticity constant of any spring $K=3k_{B}T/l^{2}$ is related to the temperature *T*, the Boltzmann constant $k_{B}$, and to the mean square bond length $l^{2}$ (we note that loop closure leads to a shrinking of bonds [34], hence $l^{2}$ is the parameter of a spanning tree of the network). Here $f_{yi}$ and $F_{yi}$ are the *y*-components of the stochastic forces and the external forces acting on the *i*th bead, respectively. Making use of the fluctuation–dissipation theorem, the random forces $f_{i}$ are connected with the dissipative friction and they are considered to be a Gaussian process, which has its first two moments written as $\langle f_{\alpha i}\left(t\right)\rangle =0$ and $\langle f_{\alpha i}\left(t\right)f_{\beta j}\left(t^{\prime }\right)\rangle =2k_{B}T\zeta \delta_{ij}\delta_{\alpha \beta }\delta \left(t-t^{\prime }\right)$ (with α and β denoting the x,y, and *z* directions). All the information about the topology of the GGS are stored in the connectivity matrix $A=A_{ij}$, which is also called the Laplacian (or Rouse) matrix [19]. This matrix is an N×N symmetric matrix, having its nondiagonal elements $A_{ij}$ equal to −1 if the the *i*th and *j*th beads are directly connected and 0 otherwise; while the diagonal elements $A_{jj}$ are equal to the number of bonds adjacent to bead *j*.

Being encouraged by the experimental techniques in [35,36,37,38,39] we study the motion of the GGS under a constant external force $F=F\cdot \Theta \left(t\right)\cdot e_{y}$ (where Θ(t) is the Heaviside step function), switched on at t=0 and acting on a single bead in the *y*-direction. After averaging over the random forces $f_{i}\left(t\right)$ and over all the beads in the GGS, the displacement is given by [20,23,27,31]

(4) $$\langle \langle Y\left(t\right)\rangle \rangle =\frac{Ft}{N\zeta }+\frac{F}{\sigma N\zeta }\sum_{n=2}^{N}\frac{1-\exp(-\sigma \lambda_{n}t)}{\lambda_{n}},$$

where $\sigma =\frac{K}{\zeta }$ is the bond rate constant. In this model, the average displacement depends only on the eigenvalues $\lambda_{n}$ of the connectivity matrix A, but not on its eigenvectors. In the case of more complex force configurations (e.g., as used for layered flows [40]), the eigenvectors are indispensable. From Equation (4), the behavior of the averaged displacement for extremely short times and for very long times becomes evident. In the limit of very short times and sufficiently large *N* one gets 〈〈Y(t)〉〉=Ft/ζ, and for very long times one obtains 〈〈Y(t)〉〉=Ft/Nζ. Thus, for very short times one observes only the motion of single beads that do not yet feel their neighbors, whereas for very long times the whole GGS diffuses, resulting in an increase of the friction from ζ to Nζ. However, in the intermediate time region there is a strong dependence on the particular topology of the GGS; the behavior of the averaged displacement will indeed depend on the eigenvalues of the matrix A. Since in this article we are mainly interested in the characteristic behavior of 〈〈Y(t)〉〉, we consider F/ζ=1 and σ=1.

In this article we are also interested in the viscoelastic properties of the polymeric structures, and we calculate the mechanical relaxation form; namely, the complex dynamic modulus $G^{*}\left(\omega \right)$ or, more exactly, its real $G^{\prime }\left(\omega \right)$ and imaginary $G^{\prime \prime }\left(\omega \right)$ components (known as the storage and the loss moduli) [41,42]. For very dilute solutions, the storage and loss moduli are given by [20]

(5) $$G^{\prime }\left(\omega \right)=\nu k_{B}T\frac{1}{N}\sum_{i=2}^{N}\frac{\omega^{2}}{\omega^{2}+{\left(2\sigma \lambda_{i}\right)}^{2}}$$

and

(6) $$G^{\prime \prime }\left(\omega \right)=\nu k_{B}T\frac{1}{N}\sum_{i=2}^{N}\frac{2\sigma \omega \lambda_{i}}{\omega^{2}+{\left(2\sigma \lambda_{i}\right)}^{2}}.$$

In (5) and (6) ν is the number of polymer segments (beads) per unit volume and, as in Equation (4), $\lambda_{i}$ are the eigenvalues of the connectivity matrix A. In these equations only the non-vanishing eigenvalues are considered, because $\lambda_{1}=0$ corresponds to the translation of the system as a whole and does not contribute to the moduli. The factor 2 in the relaxation times $\tau_{i}=1/2\sigma \lambda_{i}$ appears from the stress–stress correlations, leading to a product of two bond autocorrelation functions [43]. As in the case of monomer displacement, we are mostly interested in the slopes of $G^{\prime }\left(\omega \right)$ and $G^{\prime \prime }\left(\omega \right)$, and therefore choose $\nu k_{B}T/N=1$ and σ=1 in (5) and (6).

## 4. Results

### 4.1. Eigenvalues Spectrum

**Figure 2:**
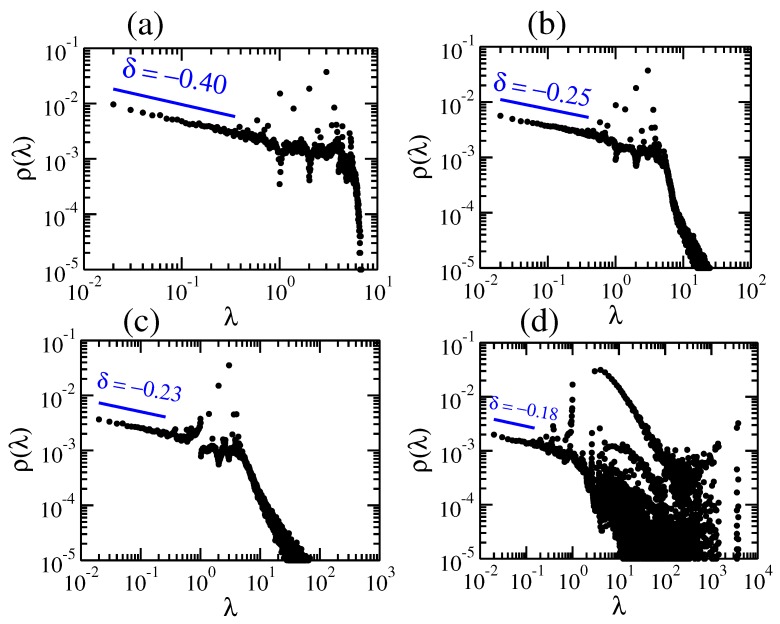
Spectral density of S=100 realizations of pDSFNs with N= 10,000 and γ=2.5 for different values of *p*: (**a**) 0.0, (**b**) 0.01, (**c**) 0.1, and (**d**) 1.0.

In Figure 2 we display the eigenvalues’ density, ρ(λ), in double logarithmic scale, for pDSFNs with N= 10,000 nodes and S=100 realizations (the Laplacian matrices A are diagonalized using subroutines of the LAPACK package [44] in FORTRAN). Here, we vary the parameter *p*, which controls bond addition to the rings, from p=0.0 (rDSFN consisting of rings) to p=1.0 (cDSFN consisting of complete graphs) for an intermediate value of the parameter γ=2.5. We note that for pDSFNs with very high γ the parameter *p* does not play an important role, since the number of rings with more than three nodes gets low. In Figure 2a one can notice a weak interplay between a single ring or chain’s spectrum, namely a continuous spectrum until λ≈4, and traces of a collection of coupled rings. Increasing the parameter *p* we get nodes with higher functionalities, which provide an increase in the magnitude of the highest eigenvalues, enlarging the width of the spectrum. This enlargement can also be understood by employing some considerations to the number of links. By increasing the parameter *p*, the number of additional links will increase; thus, the sum of all the eigenvalues will also increase: $\sum_{i}\lambda_{i}=2L$, where *L* stands for the total number of links. However, the total number of eigenvalues keeps the same, *N*, and as a consequence we expect higher eigenvalues when *p* gets higher. Even for very small values of *p*, which correspond to a small amount of additional links, we observe a clear difference from the rDSFNs (no additional internal links). This fact was also observed for another type of network: small-world networks [45]. In the region of high eigenvalues, the appearance of a power-law behaviour occurs even for very low *p*, namely p=0.01. For a higher value of the parameter (p=0.1), this behaviour gets more pronounced and the appearance of a fat tail gets more visible; see Figure 2c. For cDSFNs, which corresponds to p=1.0, the higher eigenvalues get larger and we also notice an increase in their degeneracy, as shown in Figure 2d. Notwithstanding the rich behavior of the eigenvalues’ density ρ(λ) at large λ (especially for higher *p*), the structural relaxation is related to rather low eigenvalues λ<1. In the region of low eigenvalues we obtain a power-law behavior with the exponent δ that varies from −0.4 for p=0.0 to −0.18 for p=1.0. We remark that one can define the spectral dimension $d_{s}$ based on the exponent δ by the relation $\delta =\frac{d_{s}}{2}-1$, following the pioneering work of reference [46].

### 4.2. Relaxation Dynamics

Now we consider the relaxation dynamics of pDSFNs, starting with the components of the complex dynamic modulus—the storage and the loss moduli.

**Figure 3:**
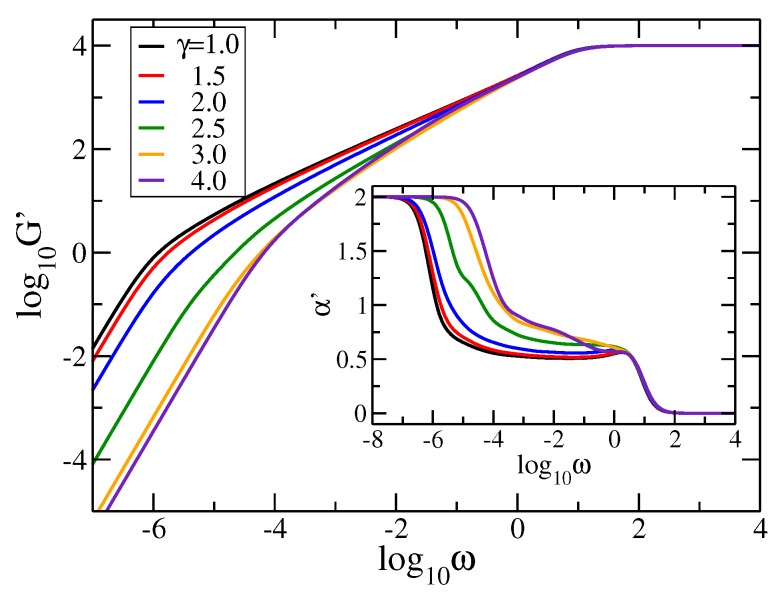
Storage modulus and the corresponding derivative (inset) for rDSFNs (p=0.0) with *N* = 10,000 and various values of γ. The frequency ω has units of σ=K/ζ.

In Figure 3 we plot in double logarithmic scale the storage modulus, Equation (5), with $\nu k_{B}T/N=1$ and σ=1, for rDSFNs (i.e., pDSFNs with p=0.0) with a fixed number of monomers, N= 10,000. Here we varied the parameter γ from 1.0 to 4.0, and for a better visualization we also display as inset figure the local derivative $\alpha^{\prime }=\frac{d(log_{10}G^{\prime })}{d(log_{10}\omega )}$ for all the curves. Immediately apparent are the limiting behaviors for very low and very high frequencies, namely power-laws with slopes 2 and 0, respectively, as it follows directly from Equation (5). In the intermediate range one notices the influence of the topology of the networks. For the studied case, p=0.0, we observe regions with almost constant slope for γ≤2.5, ranging for more than three orders of magnitude, which is due to the linear spacers of the rDSFNs. These slopes are a little bit different than the standard value of 0.5 of the linear chains [41]: $\alpha^{\prime }\approx 0.52$ for γ=1.0, $\alpha^{\prime }\approx 0.54$ for γ=1.5, $\alpha^{\prime }\approx 0.59$ for γ=2.0, and $\alpha^{\prime }\approx 0.64$ for γ=2.5 (the latter value is closely related to the spectral dimension observed in Figure 2a, bearing in mind that $\alpha^{\prime }\approx \frac{d_{s}}{2}$ [20,47]). For larger values, γ≥3.0, the region of constant slopes observed in the region $10^{-2}\leq \omega \leq 10^{0}$ disappears. This finding can be related to a growth of the number of the branches; in this case, there are nodes only with functionalities 3 and 4.

**Figure 4:**
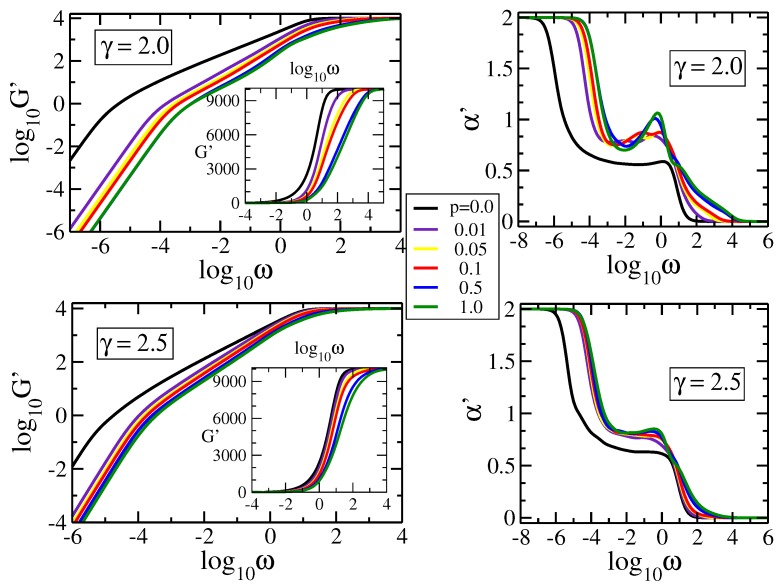
Storage modulus $G^{\prime }$ and its derivative $\alpha^{\prime }$ for pDSFNs with N= 10,000 and various values of *p* and γ, as indicated. The frequency ω has units of σ=K/ζ.

In Figure 4 we display the storage modulus, $G^{\prime }\left(\omega \right)$, for pDSFNs with N= 10,000 monomers and γ fixed to 2.0 (top row) and 2.5 (bottom row). In the right column we plot the local derivative $\alpha^{\prime }$ for all the curves from the left column. The two chosen values of γ correspond to pDSFNs that show in Figure 3a scaling behavior in the intermediate frequency domain. For these γs one obtains rDSFNs with medium-size rings, which are not as large as in the case of γ=1.0 and not as small as for γ≥3.0. In Figure 4 we monitor the influence of the parameter *p*, which was varied from 0.0 to 1.0, on the relaxation dynamics. Again, the limiting behaviors for very low and very high frequencies are well recovered. In the intermediate frequency domain one can easily notice that even for very small values of *p* (i.e., for a small amount of additional internal links between nodes from the same ring), the scaling behavior observed for rDSFNs (p=0) vanishes. It is replaced with a nonmonotonous behavior, which was also observed for some fractal polymers [48], or with another slope. In particular, for γ=2.0 and p=0.01 we notice an almost constant slope $\alpha^{\prime }\approx 0.77$ in the frequency range $10^{-3.0}\leq \omega \leq 10^{-1.5}$. For γ=2.5 the constant slope is maintained for all the values of *p*, but with slightly different values for the exponent $\alpha^{\prime }$, varying between 0.77 and 0.82. For γ=2.5 the difference between curves with different non-zero *p* is less prominent, due to a smaller amount of possible additional internal links (the differences are more visible in semi-logarithmic scales; see the insets of Figure 4). This statement will become more evident when the loss modulus is considered.

**Figure 5:**
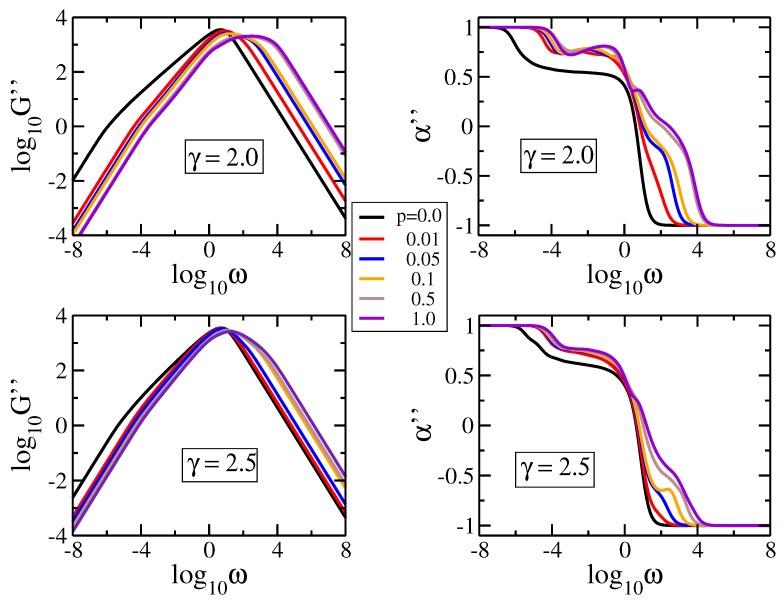
Loss modulus and its derivative for pDSFNs with N= 10,000 and various values of *p*, having γ=2.0 and 2.5. The frequency ω has units of σ=K/ζ.

Now we turn our attention to the influence of *p* on the loss modulus, Equation (6). In Figure 5 we display in double logarithmic scale the modulus and its the derivative $\alpha^{\prime \prime }=\frac{d(log_{10}G^{\prime \prime })}{d(log_{10}\omega )}$ for γ=2.0 and 2.5. Here we set $\nu k_{B}T/N=1$ and σ=1. The universal structure-independent limiting behaviors for very low and very high frequencies (namely power-laws with slopes −1 and 1, respectively) follow directly from Equation (6). For each value of γ we choose the same *p*-values as in Figure 4, from 0.0 to 1.0. As previously observed, even for small values of *p* the behavior changes drastically when the parameter *p* is switched on. Additionally, for higher γ the size of the rings gets smaller, meaning that the number of possible additional links decreases; thus, there are only slight differences between various p>0.01-values. For pDSFNs with γ=2.5 we observe scaling in the intermediate frequency region for all the values of *p*, while for γ=2.0 we get a region of constant slope $\alpha^{\prime \prime }\approx 0.75$ for pDSFNs with the parameter *p* equal to 0.01. From Figure 5 a shift towards a higher frequency region is evident when *p* gets higher, which fades away by increasing the parameter γ. These findings can be understood by considering the average number of rings, which can be written as <g> = (N−1)/(<n>−1), where <n> is the average size of rings. The last quantity follows from Equation (2), and it can be written in the thermodynamic limit (N→∞) based on the Riemann zeta function [49] as <n> = (ζ(γ−1)−1)/(ζ(γ)−1). This equation provides a finite and relatively small value of <n> ≈ 4.72 for γ=2.5, but for γ=2.0 the average size of rings <n> grows logarithmically with *N*. Thus, for γ=2.5 there is a high number of connected small-size rings, whereas for γ=2.0 one has a collection of rather large rings. Hence, in the case of large rings (γ=2.0) more bonds to them can be added, leading to a more significant variation of the structures’ size, which is reflected in the more significant shift of the moduli by varying *p*.

**Figure 6:**
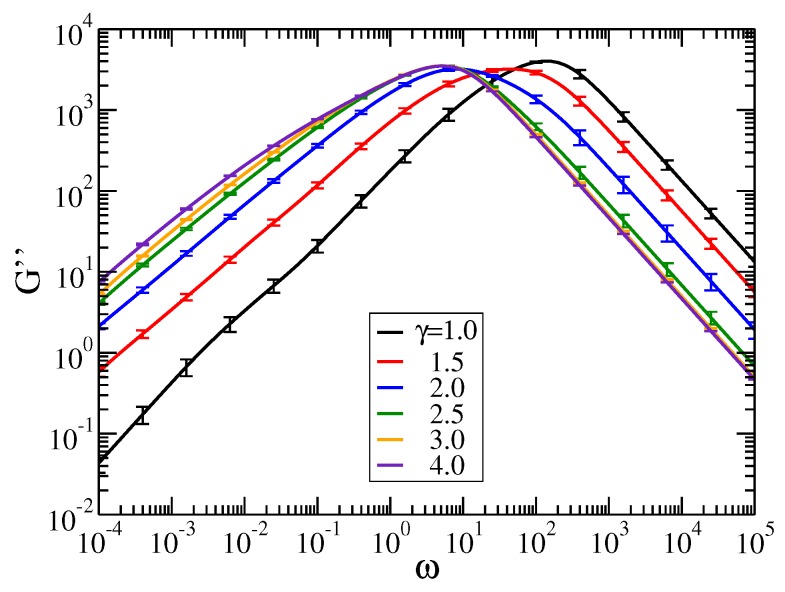
Loss modulus for pDSFNs with N= 10,000, p=0.01, and various values of γ. The frequency ω has units of σ=K/ζ. Error bars show the standard errors.

As we have observed in Figure 4 and Figure 5, the value p=0.01 leads to a transition to a new characteristic behavior. Therefore, in Figure 6 we consider the loss modulus, Equation (6), for pDSFNs with p=0.01 and γ ranging from 1.0 to 4.0. In the intermediate range of frequencies, where the topology of the networks plays an important role, we observe a region with almost constant slope for γ=2.0, which is less pronounced for other values of γ. The final important point in the discussion of moduli is addressed to the error bars. As can be inferred from Figure 6, for γ equal to 2.0 and higher, the standard errors for frequencies ω/σ>1 are comparable with the line thickness, allowing for a quite good estimation of the possible slopes in that region. On the other hand, for γ=1.0 the error bars are quite large. This observation corresponds to the (logarithmically) divergent behavior of the normalization of distribution $p_{k}$; see Equation (2). For the intrasegmental frequency region ω/σ≫1 we find higher standard deviations of the moduli, reflecting the rich behavior of the eigenvalues’ density ρ(λ) at large λ; see Figure 2. However, aiming to study here the structural relaxation of the networks, this region is a minor focus of this work.

**Figure 7:**
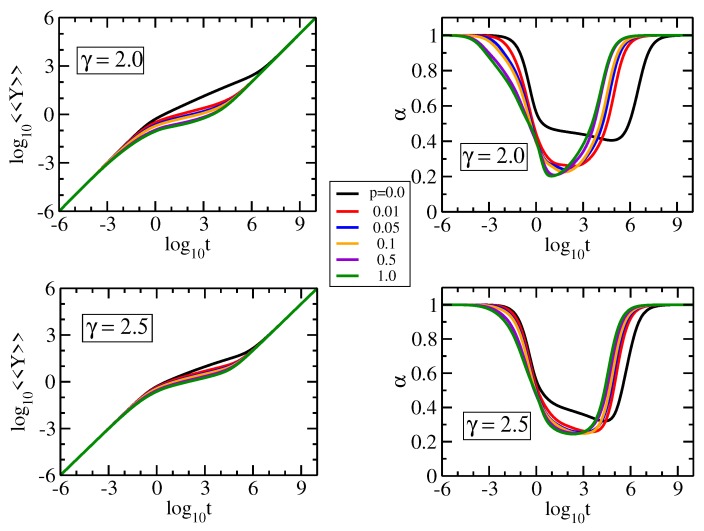
Average monomer displacement and its derivative for pDSFNs with N= 10,000 and various values of *p* and γ, as indicated. The time *t* has units of 1/σ=ζ/K.

The characteristic behaviors observed in the mechanical relaxation are also reflected in other dynamical properties, as we proceed to show by considering the average monomer displacement 〈〈Y(t)〉〉, Equation (4). In Figure 7 we show in double logarithmic scale 〈〈Y(t)〉〉 with F/ζ=1 and σ=1, for pDSFNs of γ=2.0 and 2.5. In the left panels we display the local derivative $\alpha =\frac{d(log_{10}\langle \langle Y\rangle \rangle )}{d(log_{10}t)}$ of the curves plotted in the right panels. We fixed the parameters (N,S) to (10,000, 100) and we varied the parameter *p* from 0.0 to 1.0. Immediately apparent for all panels are the limiting behaviors in the region of very short and very long times; namely, a linear time-dependence, 〈〈Y(t)〉〉∝t, as discussed in Equation (4). Already for small values of p>0 a new scaling behavior (related to that observed in the mechnical relaxation) was encountered; namely (γ,p)=(2.0,0.01) with α≈0.26. Remarkably, for γ=2.5 we observe a more pronounced slope region of almost two orders of magnitude, α≈0.25. The constant slope of 〈〈Y(t)〉〉 in the intermediate time region corresponds to the spectral dimension, $\alpha =1-d_{s}/2$; see [20] for more details. The dependence on *p* gets lower by increasing γ, because the networks have a small amount of rings with more than three nodes, which do not have an internal bond. For an illustration of this statement see the corresponding typical realization in Figure 1f.

## 5. Conclusions

In this paper we have studied a new kind of polymeric networks that are the dual structures of treelike scale-free networks. The dual patterns are realized based on sequentially connected rings whose size follows a scale-free degree distribution [15]. Hence the topology of these networks varies with the power-law exponent γ of the scale-free distribution. For small values of γ we get with high probability some connected rings which have a very large size (similar to the hubs from treelike scale-free networks) and for very high γs we obtain a large amount of linear segments and small rings. Furthermore, we have also considered a small-world like [23,29,30] transition of the sequentially attached rings towards complete graphs. In doing so, we have added links with probability *p* to nodes from the same rings. In the limiting case, p=1.0, we have obtained networks of sequentially connected complete graphs.

The relaxation dynamics of these networks is studied on the mechanical relaxation moduli and the average monomer displacement, employing the generalized Gaussian structures’ framework [20]. Addition of bonds into the sequentially growing rings plays a crucial role in the dynamical behavior of the polymeric networks. Already for a low probability of having a bond, p=0.01, we have encountered a new scaling behavior which is persistent for several values of the tuple (γ,p). So, for the mechanical relaxation moduli we find characteristic exponents with values between 0.75 and 0.82, which are then reflected in the time behavior of the monomer displacement characterized by the exponents close to the value 0.25. Thus, the addition of bonds leads to a slowing down on the dynamics.

Our findings can be helpful for studies of supramacromolecular complexes, such as core–shell nanogel systems [10]. From the theoretical point of view, further extensions of the model through inclusion of excluded volume and hydrodynamic interactions can be of much interest. Such extensions will allow for a direct parametrization of the real polymeric network systems. Moreover, so far we have considered structure-averaged global behavior of the networks that can be computed based only on the eigenvalues of the connectivity matrices. In order to distinguish, for example, linear dangling segments, consideration of the local re-orientation properties (that are captured, e.g., in the NMR relaxation experiments [50,51]) will be of much help. Computation of the related characteristics will additionally require knowledge of the eigenvectors of the connectivity matrices [52]. Additionally, not only the averaged quantities, but their distributions will be an interesting topic for future work.

## Abbreviations

The following abbreviations are used in this manuscript:

GGS	generalised Gaussian structures
rDSFNs	ring-based dual scale-free networks
cDSFNs	complete-graph based dual scale-free networks
pDSFNs	sequentially growing partially dual scale-free networks
